# A Coupled Refined Model of Atomistic and Continuum Parameters of Diatomic Covalent Bonds

**DOI:** 10.3390/nano16060347

**Published:** 2026-03-12

**Authors:** Oleksandr Hondliakh, Sergiy Antonyuk, Marc Weirich, Simon Paas

**Affiliations:** Institute of Particle Process Engineering, RPTU University Kaiserslautern-Landau, 67663 Kaiserslautern, Germany; sergiy.antonyuk@mv.rptu.de (S.A.); marc.weirich@mv.rptu.de (M.W.); simon.paas@mv.rptu.de (S.P.)

**Keywords:** carbon, covalent bond, molecular mechanics, structural mechanics, nanotubes, Poisson’s ratio, effective bond diameter

## Abstract

This study addresses the challenge of consistently transferring atomistic parameters of the C–C bond into phenomenological material characteristics within the framework of continuum mechanics. Particular attention is given to determining the effective transverse diameter of the covalent C–C bond in carbon nanostructures. The dependence of this diameter on Poisson’s ratio ν is examined, and the influence of the interatomic stiffness constants $k_{r},k_{\theta }\mathrm{and}k_{\tau }$ is systematically analyzed. Classical representative-volume models of the C–C bond based on the Euler–Bernoulli beam hypothesis violate thermodynamic stability conditions and lead to nonphysical Poisson’s ratio values exceeding 0.5, due to the neglect of shear deformation effects. To overcome this limitation, an approach based on Timoshenko beam theory is proposed, accounting for both bending and shear deformations. This approach enables estimation of energetically equivalent states between the phenomenological representative volume and the corresponding atomistic C–C bond model. As a result, a sixth-order algebraic equation is derived linking the effective bond diameter, the Poisson’s ratio, and the molecular mechanics force constants. Analysis of this equation reveals a narrow range of effective bond diameters and Poisson’s ratios for which thermodynamic stability conditions are satisfied. Within this range, physically consistent macroscopic material parameters can be directly expressed in terms of atomistic force constants.

## 1. Introduction

Carbon nanostructures, such as nanotubes and graphene, possess unique mechanical properties—high stiffness, strength, and resistance to deformation. Their behavior at the nanoscale is typically described using molecular dynamics (MD) methods, which allow for the explicit consideration of atomic interactions through empirical potentials, such as Morse or Lennard-Jones formulations.

However, the direct application of MD models to the description of large-scale systems is associated with significant computational costs due to the need to simulate a representative volume of material containing a relatively small number of molecules or atoms, as well as to the limitation of modeling processes over very short time intervals (from picoseconds to nanoseconds) [1].

This significantly complicates the macroscopic modeling of nanomodified materials and structures derived from them and, in some cases, renders such modeling impossible.

In this context, there is a need to develop specialized multiscale models that allow for a consistent transition from atomistic mechanics parameters (bond stiffness under tension, bending, and torsion, potential parameters) to phenomenological parameters of solid mechanics (elastic modulus and Poisson’s ratio, shear modulus, plasticity, etc.). Such a transition requires the development of specialized models in which an individual chemical bond is interpreted as a mechanical element with angular, radial, and torsional stiffness. In this regard, the question arises regarding the correct choice of geometric parameters, in particular the effective diameter of the C–C chemical bond, which allows the reduction of the interatomic interaction equations to the classical equations of continuum mechanics.

The main difficulty lies in the fact that the formulas of structural mechanics include Poisson’s ratio, whereas at the level of an individual chemical bond, this parameter is not explicitly defined. As a result, traditional methods for determining the energetically equivalent mechanical properties of an effective rod depend on an arbitrarily chosen value of Poisson’s ratio ν. Moreover, the direct application of classical beam theory equations to carbon nanostructures frequently results in nonphysical values of Poisson’s ratio, exceeding the upper limit of ν = 0.5 permitted for isotropic continuum materials.

Thus, a contradiction arises when directly transferring the molecular dynamics bond stiffnesses of the C–C bond into the classical beam theory equations. From the well-known relations that energetically link the force constant of molecular mechanics $k_{r},k_{\theta }\;и\;k_{\tau }$ with the physico-mechanical characteristics of a rod with a circular cross-section of diameter d within the straight-normal (Euler–Bernoulli) hypothesis [2] ($k_{r}=\frac{E\pi d^{2}}{4L}$, $k_{\theta }=\;\frac{E\pi d^{4}}{64L}$, and $k_{\tau }=\;\frac{G\pi d^{4}}{32L}$), it follows that $d=4\sqrt{\frac{k_{\theta }}{k_{r}}}$. After substituting the known values $k_{r}=\;651.694\frac{nN}{nm},\;{\;k}_{\tau }\;=\;0.278\;\frac{nN\;nm}{{rad}^{2}}{,\;k}_{\theta }=\;0.901\;\frac{nN\;nm}{{rad}^{2}}$ for the C–C covalent bond, we obtain d≈0.147 nm, E ≈5.49 TPa, and G ≈0.871 TPa. At the same time, if Poisson’s ratio ν is determined from the known relations for an isotropic body, as $\nu =\frac{E}{2G}-1$, we obtain $\nu =\;\frac{5.49}{2\times 0.871}-1\approx 2.15\;>0.5$, which is unphysical, since it violates the thermodynamic stability conditions for the continuum mechanics model of isotropic bodies (as volumetric compression would lead to a negative energy increment).

Thus, the use of classical formulas for tension, bending, and torsion of a beam to determine the effective physical parameters of the C–C covalent bond can lead to nonphysical values of Poisson’s ratio, thereby highlighting the need to address the problem of a consistent and physically justified coupling between atomistic and continuum models.

The aim of this work is to develop a model that provides a physically consistent and thermodynamically admissible link between the atomistic molecular-mechanics parameters of the C–C bond and the phenomenological parameters of continuum mechanics (E, G, ν). We show that there exists a specific range of variation in the effective C–C bond diameter within which the conditions of thermodynamic stability are satisfied when transitioning from molecular-mechanics equations to phenomenological equations of solid mechanics. This eliminates the problem of obtaining nonphysical values of Poisson’s ratio and allows the mean diameter within this range to be considered as a universal bond characteristic, ensuring a consistent transition from the atomistic description of the C–C bond to its continuum analogue. The model is not intended to replace quantum-mechanical or atomistic simulations. Instead, it enables the derivation of effective geometric and elastic parameters of C–C bonds that can be directly implemented in continuum-based numerical methods.

## 2. Materials and Methods

### 2.1. State of the Art in Modeling the Effective Mechanical C–C Bond Properties

The carbon–carbon covalent (C–C) bond is a key element in the structure of carbon materials. Its electron density determines the spatial configuration, which in turn governs the mechanical and physical properties of nanostructures, including graphene, carbon nanotubes, and diamond-like materials. Research aimed at determining the macroscopic characteristics of C–C bonds from molecular mechanics parameters began developing actively in the late 1980s and received particular attention after the discovery of carbon nanotubes [3]. The main focus was on establishing the relationship between empirical interatomic potentials and effective elastic constants.

To date, several key research directions have emerged in the literature, such as:

- the development and refinement of interatomic interaction potentials [4,5,6,7,8,9];
- coupling molecular dynamics models with continuum models (beam and shell analogues of bonds) [10,11,12,13,14,15,16];
- the experimental validation (measurements of strength and elasticity of nanotubes and graphene) [4,17,18,19];
- the application of quantum-chemical and density functional theory (DFT) methods for refining C–C bond parameters [20,21,22];
- the development of multiscale theories integrating data from different approaches [23,24,25,26].

The determination of macroscopic mechanical properties of carbon nanostructures, such as carbon nanotubes and graphene, is directly related to the accurate description of the properties and geometric parameters of the C–C covalent bond. To date, one of the key challenges remains the transition of atomistic molecular mechanics parameters (bond stiffnesses, potential functions) to macroscopic continuum mechanics parameters (Young’s modulus, Poisson’s ratio, shear modulus).

Early works [5,6,27] laid the foundation for the theory of interatomic interaction potentials describing atomic interactions. Based on these studies, REBO (Reactive Empirical Bond-Order) potentials [7] and their AIREBO [9] modifications were developed, which are widely used in molecular dynamics simulations [8].

These models allow the determination of microscopic bond stiffnesses. However, when these stiffnesses are transferred to continuum-scale descriptions (e.g., beam or shell analogues of covalent bonds), additional geometric assumptions must be introduced.

Classical force-field methods (e.g., UFF [28,29], AMBER [30,31], CHARMM [32,33], MM2 [34,35]) are interpolation-based energy potentials that represent the system’s total energy as a sum of contributions from bond stretching, valence angle bending, torsional angles, and noncovalent interactions (Lennard-Jones and Coulomb forces). They are widely used in molecular mechanics and molecular dynamics to model organic molecules, biomolecules, and polymers. A comparison of these methods is provided in [36].

These force fields are typically formulated within the framework of nonreactive molecular mechanics, where the total potential energy is expressed as a sum of harmonic bond stretching, angle bending, torsional terms, as well as noncovalent Lennard-Jones and electrostatic interactions. Their main applications include organic molecules, polymers, biomolecules, and soft condensed matter systems. These methods have clear advantages, such as high computational efficiency, a broad database of parameterizations for organic and biomolecular systems, and straightforward implementation in standard MD packages. Although these models reproduce equilibrium geometries and vibrational spectra with reasonable accuracy under near-equilibrium conditions, they were not originally parameterized to reproduce the elastic constants of sp^2^ carbon bonds, such as in graphene or carbon nanotubes. Specifically, they: do not explicitly account for the dependence of bond stiffness on the local atomic environment; assume a fixed bond topology and therefore cannot describe rehybridization or bond breaking; and require additional geometric assumptions to transition from atomistic bond stiffness constants to continuum-scale elastic moduli.

In contrast, reactive bond-order potentials such as REBO, AIREBO, Tersoff, and ReaxFF incorporate an explicit dependence of bond stiffness on the local atomic environment and allow for bond rearrangement and rehybridization. For this reason, they provide a physically more consistent atomistic description of stiffness redistribution in sp^2^ carbon systems. However, even in this case, the transition from atomistic stiffness constants to continuum elastic moduli is not automatic and still requires the introduction of structural-mechanical analogies (beam or bar representations).

In the present work, the Morse potential is adopted as a reference interaction model for the C–C bond [6,16,24]. The rationale for this choice is fourfold.

First, the Morse potential provides an anharmonic energy–distance relationship with a physically meaningful dissociation energy and equilibrium bond length, allowing a consistent derivation of the radial stiffness from the second derivative of the potential at equilibrium.

Second, its analytical form ensures transparent parameter identification and a direct linkage between microscopic energy parameters and continuum elastic constants.

Third, unlike complex bond-order potentials, the Morse potential enables a controlled and explicit separation between atomistic energy description and continuum geometric representation, which is essential for analyzing the role of the effective bond diameter in structural-mechanical mapping.

Fourth, the Morse potential possesses a well-established quantum-mechanical foundation. It represents one of the few analytically solvable models of the one-dimensional stationary Schrödinger equation for a diatomic system.

As a consequence, its parameters—the dissociation energy $D_{e}$ , equilibrium bond length $r_{0}$ , and stiffness parameter α —are directly related to vibrational energy levels and spectroscopic constants. This analytical solvability ensures that the derived bond stiffness is not merely an empirical fitting parameter, but is consistently connected to the quantum-mechanical description of the covalent bond. Therefore, the Morse potential is employed here not as a universal reactive model, but as a physically interpretable and analytically tractable basis for establishing a consistent multiscale transition.

In particular, the Morse functional provides an explicit expression for the interatomic force and its linearized stiffness at equilibrium through the second derivative of the potential. This yields a well-defined axial (radial) stiffness of the C–C bond in purely energetic terms, without invoking any geometric assumptions beyond the equilibrium bond length.

However, the transition from this atomistic description to a structural-mechanical representation of a nanostructure requires embedding the bond into a three-dimensional continuum framework. In beam or bar analogies commonly used for graphene and carbon nanotubes, axial, bending, and torsional rigidities must be expressed through continuum relations involving cross-sectional properties (area and second moment of area). These quantities cannot be obtained from the interatomic potential itself, since the potential defines only the energy as a function of interatomic distance and local environment, but does not prescribe a physical cross-section of the bond.

Thus, independently of whether classical nonreactive force fields, reactive bond-order potentials, or simplified anharmonic potentials such as Morse are used, the continuum mapping step introduces an additional geometric parameter—the so-called effective C–C bond diameter.

This parameter is not uniquely defined at the atomistic level and emerges only within the structural-mechanical interpretation of the bond.

However, the literature shows significant discrepancies in the values of the effective diameter of the C–C bond, which lead to substantial variations in the calculated effective elastic modulus and Poisson’s ratio of representative C–C bond volumes. In particular, the effective values of Young’s modulus calculated based on REBO and AIREBO potentials range from 0.9 to 1.3 TPa [37], while the Poisson’s ratios range from 0.15 to 0.45. At the same time, the use of structural-mechanical analogies sometimes leads to nonphysical values of ν > 0.5 [38,39,40].

Recent studies [39,41] show that when choosing an effective diameter d for the C–C bond in the range of 0.04 nm to 0.10 nm, significant discrepancies can arise in the calculation of Poisson’s ratio ν. For example, when using d ≥ 0.1 nm, the values of ν can exceed 0.5, which is unphysical from the perspective of structural mechanics, as it violates the condition of thermodynamic stability. With a more precise consideration of molecular-mechanical parameters and a refined value range of d ≈ 0.076 nm–0.09 nm, the resulting values of ν return to the physically permissible range (0 < ν < 0.5).

Studies addressing this problem [4] have shown that different potentials yield significantly varying values of elastic moduli if the bond diameter correction is not taken into account [23]. Particular attention in the literature is paid to the fact that standard beam and bar theory equations, when applied directly to a covalent bond, can lead to unphysical results if a diameter value of around 0.1 nm is used without correction. Therefore, the commonly accepted bond diameter d = 0.147 nm should not be used without appropriate corrections [17,39].

Therefore, the present work does not propose an alternative interatomic potential. Instead, it focuses on the consistent identification of continuum-level bond parameters based on atomistic stiffness characteristics, eliminating the ambiguity associated with the effective bond diameter. In this sense, the central objective is to establish a mechanically and thermodynamically consistent bridge between atomistic interaction models and structural mechanics representations.

Thus, the key issue is not simply the numerical choice of the effective diameter, but its physically consistent determination within a multiscale formulation that ensures compatibility between atomistic stiffness parameters and the requirements of thermodynamic stability at the continuum level.

### 2.2. Selection of Calculation Schemes for C–C Bond

The method proposed in this work for determining the physics-mechanical characteristics of a C–C covalent bond between two carbon atoms consists of the following steps:

- Determination of the effective C–C bond diameter, taking shear deformation into account, using a refined Timoshenko beam model;
- Determination of the effective Poisson’s ratio;
- Determination of the effective Young’s modulus based on the Morse potential;
- Determination of the effective shear modulus of the C–C bond.

Let us consider each of these points separately.

Accounting for shear deformation using the Timoshenko theory when modeling the C–C covalent bond as a beam model is necessary due to the fundamental difference between the assumptions of classical Euler–Bernoulli bending theory and the physical reality of interatomic interactions at the nanoscale.

Classical Euler–Bernoulli beam theory is based on two key assumptions:

- The plane section hypothesis: It is assumed that the beam’s cross-sections remain plane and perpendicular to the neutral axis during bending.
- Neglect of transverse shear: This model assumes infinite material stiffness with respect to transverse shear (G→∞).

These assumptions work well only for “very long” and “very thin” beams, where the primary contribution to deformation comes from pure bending, and shear deformation is negligibly small. In the case of modeling an individual C–C covalent bond as a beam in a continuum model (for example, to calculate the elastic properties of carbon nanotubes), the C–C bond has a length of approximately 0.142 nm, and the effective “bond diameter”, determined according to the plane section hypothesis ($d_{\theta }\approx 4\sqrt{\frac{k_{\theta }}{k_{r}}}=0.147\mathrm{nm}$ ), is not negligibly small compared to the bond length.

In this context, the C–C bond, according to the classification of beam theories, can be considered a geometrically “short” or “thick” beam. Therefore, the use of Euler–Bernoulli theory in this case leads to an artificially overestimated bond diameter and an underestimated bond elastic modulus as a result of compensating for the neglected shear deformation. In addition, when real atomic structures (e.g., molecules or nanotubes) bend or vibrate, the angle between bonds can change significantly, which further necessitates accounting for transverse shear deformation in the continuum model.

Therefore, in this work, the Timoshenko model is adopted as the fundamental hypothesis for the transition from the atomic model to the continuum, and its advantages can be summarized as follows:

- Timoshenko theory, by introducing the shear angle as an independent parameter, removes the assumption of cross-section perpendicularity to the neutral axis and accounts for both bending deformation and transverse shear deformation independently.
- The total potential deformation energy in the Timoshenko model includes not only the bending energy but also the shear energy. In this case, the Timoshenko model provides a more physically adequate representation of the behavior of continuum bond models when simulating the actual behavior of atoms and bonds, especially when analyzing local bending and high-frequency vibrations (when the wavelengths are comparable to interatomic distances).
- Furthermore, accounting for shear strain plays a significant role in the analysis of high-frequency vibrational behavior (for example, when determining the natural modes and frequencies of vibrations of nanostructures).

In addition, the Timoshenko model allows for a more adequate description of local stiffness variations caused by defects (e.g., Stone–Wales defects) or changes in the orientation of C–C bonds, which is important for a more accurate determination of the strength characteristics of nanostructures.

Thus, accounting for shear using the Timoshenko theory is not strictly necessary for determining the intrinsic diameter of the C–C bond but is required for the correct application of continuum models when calculating the mechanical and dynamic properties of systems composed of these bonds (e.g., carbon nanotubes), where the classical, simplified Euler–Bernoulli theory produces significant errors due to the considerable influence of transverse shear and can lead to unphysical results when obtaining effective physics-mechanical and geometric parameters.

### 2.3. C–C Bond Shear Effect

The simplest method for incorporating the shear effect was proposed in [42], where a shear coefficient was introduced into the bending equations of the Timoshenko beam.

There are several methods for determining the shear coefficient K. In general, it should satisfy the following condition:

(1) $$\int_{S}\sigma_{\tau }dA=K\;A\;G\;\varphi ,$$

where $\sigma_{\tau }$ is the transverse shear stress acting in the beam, A is the cross-sectional area of the beam, G is the shear modulus of the beam material, and φ is the rotation angle of the cross-section normal during deformation.

According to [43], the shear coefficient can be expressed by an equation that depends on the Poisson’s ratio and the geometric parameters of the beam’s cross-section as follows:

(2) $$K=\;\frac{2\left(1+\nu \right)I_{x}}{\frac{\nu (I_{y}-I_{x})}{2}-\;\frac{A}{I_{x}}\iint x\left(\chi +xy^{2}\right)dxdy},$$

where χ(x,y,ν) is a harmonic function depending on the coordinates x,y of the beam’s cross-section and the Poisson’s ratio ν [43], $I_{x}=\iint y^{2}dxdy$ is the moment of inertia of the cross-section about the y-axis, and $I_{y}=\iint x^{2}dxdy$ is the moment of inertia of the cross-section about the x-axis.

For a beam with a solid circular cross-section, the shear coefficient K is equal to

(3) $$K=\;\frac{6(1+\nu )}{\left(7+6\nu \right)},$$

and for a solid rectangular cross-section, it is equal to

(4) $$K=\;\frac{10(1+\nu )}{12+11\nu }.$$

In this case, it does not depend on the ratio of the beam’s width to its height [43].

The total steric potential energy of a diatomic C–C system is determined by the spatial arrangement of the atoms. For small linear elastic deformations, neglecting electrostatic interactions and the energy associated with nonbonded van der Waals forces, it can be expressed as [2,11]:

(5) $$U_{total}=U_{r}+U_{\theta }+U_{\varphi }+U_{\omega }=U_{r}+U_{\theta }+U_{\tau }\;,$$

where:

$U_{r}=\frac{1}{2}k_{r}{\left(\Delta r\right)}^{2}$—energy associated with bond tension;
$U_{\theta }=\frac{1}{2}k_{\theta }{\left(\Delta \theta \right)}^{2}$—energy associated with bond angle change during bending;
$U_{\tau }=U_{\varphi }+U_{\omega }=\frac{1}{2}k_{\tau }{\left(\Delta \varphi \right)}^{2}$—energy accounting for total torsion under the dihedral angle $U_{\varphi }$ and out-of-plane torsion energy $U_{\omega }$.

**Figure 1:**
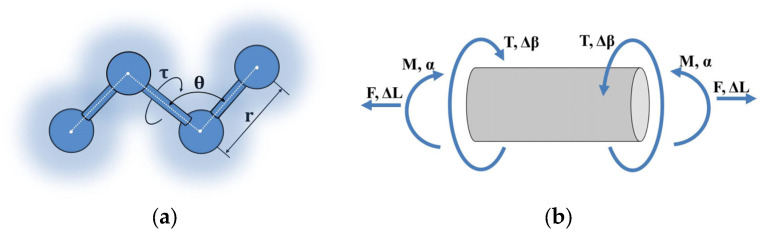
Interaction schemes: (**a**) Interatomic; (**b**) Beam analogue.

The force constants $k_{r}$, $k_{\theta }$ and $k_{\tau }$ represent the bond’s resistance to tension, bending, and torsion, respectively. Δr, Δθ and Δφ correspond to the increments of longitudinal displacement under tension, angle changes during bending, and bond torsion, respectively. A schematic representation of the interatomic interactions is shown in Figure 1a.

Following the procedure described in [2], we write the expressions for determining the potential energy of the beam for tension, bending, and torsion (Figure 1b):

(6) $$U_{F}=\frac{1}{2}\;\int_{0}^{L}\frac{F^{2}}{EA}dL=\;\frac{1}{2}\;\frac{EA}{L}{\left(\Delta L\right)}^{2}\;-\;tension,$$

(7) $$U_{M}=\frac{1}{2}\;\int_{0}^{L}\frac{M^{2}}{EI}dL=\;\frac{1}{2}\;\frac{EI}{L}{\left(2\alpha \right)}^{2}\;-\;bending,$$

(8) $$U_{T}=\frac{1}{2}\;\int_{0}^{L}\frac{T^{2}}{GJ}dL=\;\frac{1}{2}\;\frac{GJ}{L}{\left(\Delta \beta \right)}^{2}\;-\;\;torsion,$$

where E—Young’s modulus; G—shear modulus; A—cross-sectional area; L—beam length; $I=\frac{\pi d^{4}}{64}$—moment of inertia; $J=\frac{\pi d^{4}}{32}$—polar moment of inertia of a circular cross-section beam; ∆L, α, and ∆β—increment of length, bending angle, and increment of torsion angle, respectively; and d—effective bond beam diameter, nm.

By comparing the molecular mechanics equations with Equations (6)–(8) of structural mechanics, we obtain the well-known relations [2]:

(9) $$\frac{EA}{L}=\;k_{r}\;,\;\;\;\;\frac{EI}{L}=\;k_{\theta },\;\;\;\;\frac{GJ}{L}=\;k_{\tau }\;$$

from which it follows that

(10) $$E=\frac{\;4k_{r}L}{\;\;\pi d^{2}}\;\mathrm{and}\;G=\frac{\;32k_{\tau }L}{\;\;\pi d^{4}}.$$

According to [44,45], the bending stiffness of the effective representative beam volume, accounting for the correction for the shear coefficient K in Equation (3), is given by [42]:

(11) $${К}_{bend}=\frac{\;EI}{\;\;L}\;\frac{\;4+ф}{\;\;1+ф},$$

(12) $$where\;ф=\frac{\;12EI}{\;\;GA_{k}L^{2}}-shear\;deformation\;constant\;with$$

(13) $$A_{k}=\;\frac{\;A}{\;\;K}\;=\;A\;\frac{\left(7+6\nu \right)}{6(1+\nu )}.$$

Taking into account relations (10), we obtain the expression for the shear deformation constant ф:

(14) $$ф=\frac{9\;k_{r}\;d^{4}\;(1+\nu )}{16{\;k_{\tau }\;L}^{2}\;\left(7+6\nu \right)\;}.$$

By equating the bending stiffness of the effective representative bond volume (11) to the molecular mechanics force constant for bond bending (${К}_{bend}=\;k_{\theta }$), and substituting (14) and (10) into (11), we obtain the following relation, which establishes the connection between the effective diameter d and Poisson’s ratio ν through the molecular mechanics force constants:

(15) $$k_{\theta }=\frac{\;\;k_{r}d^{2}}{16}\;\frac{\;\;448\;{k_{\tau }\;L}^{2}\;+9{\;k}_{r}d^{4}+384\;{k_{\tau }\;L}^{2}\;\nu +9\;{\;k}_{r}\;d^{4}\nu }{\;{112\;k_{\tau }L}^{2}\;\;+\;\;{9\;k}_{r}d^{4}+96{\;k_{\tau }L}^{2}\;\nu +{9\;k}_{r}\;d^{4}\nu \;}.$$

After combining like terms in (15), we obtain a sixth-order algebraic equation containing nonzero coefficients only for even powers of d, or, in other words, a bicubic equation with respect to the sought diameter d, namely:

(16) $${F\left(\nu ,d\right)=9{{\;k}_{r}k}_{r}\left(1+\nu \right)d^{6}-{144k}_{r}\;k_{\theta }\left(1+\nu \right)d^{4}+64\;k}_{r}\;{k_{\tau }\;L}^{2}\left(7+6\;\nu \right)d^{2}-256{\;k}_{\tau }k_{\theta }\;L^{2}\left(7+6\;\nu \right)=0.$$

Similarly, we obtain the equation for calculating Poisson’s ratio ν:

(17) $$\nu =\frac{\;-\;9{k_{r}k}_{r}d^{6}+\;{144\;k}_{r}\;k_{\theta }d^{4}\;-\;448\;\left[k_{r}\;{k_{\tau }\;L}^{2}d^{2}-4k_{\tau }k_{\theta }\;L^{2}\right]\;}{9{k_{r}k}_{r}d^{6}-\;{144\;k}_{r}\;k_{\theta }d^{4}+\;384\;[k_{r}\;{k_{\tau }\;L}^{2}d^{2}-4k_{\tau }k_{\theta }\;L^{2}]}.$$

In [42], based on the same methodology, an expression for $k_{\tau }$ was obtained, similar to expression (15), namely:

(18) $$k_{\tau }=\frac{\;\;{\pi \;k}_{r}d^{2}}{16\pi }\;\frac{\;\;448\;{k_{\theta }\;L}^{2}\pi^{2}+384\;L^{2}\pi k_{\theta }\nu +9{\;k}_{r}\pi^{2}d^{4}+9\;{\;k}_{r}\pi^{2}d^{4}\nu }{\;{112k_{\theta }\;L}^{2}\pi^{2}+96\;L^{2}{\pi^{2}k}_{\theta }\nu \;\;+\;\;{9\;k}_{r}d^{4}\pi^{2}+{9\;k}_{r}d^{4}\pi^{2}\nu \;}.$$

**Figure 2:**
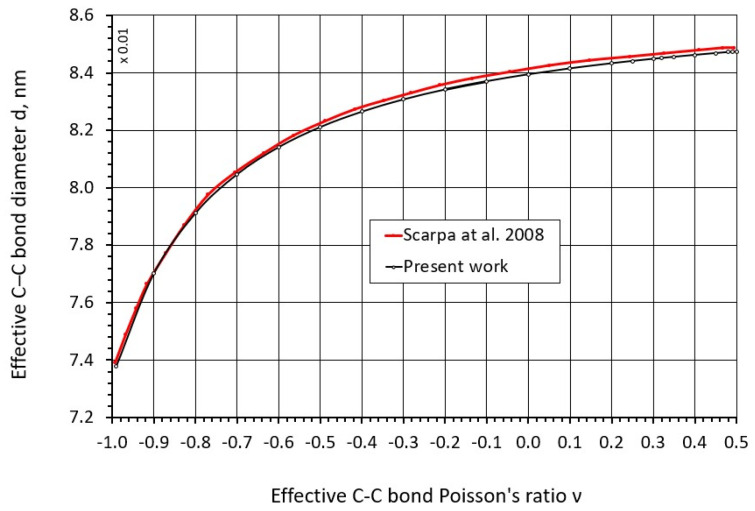
Dependence of the C–C bond diameter d on Poisson’s ratio ν [42].

Equation (16), after a change in variables, can be reduced to an algebraic cubic equation. Solving it using Cardano’s method with the force constants corresponding to the C–C bond [2] ($k_{r}=651.694\frac{nN}{nm},k_{\theta }=0.875\frac{nN\;nm}{{rad}^{2}},k_{\tau }=0.278\frac{nN\;nm}{{rad}^{2}}$), we obtain the curve of the C–C bond diameter $d_{c-c}$ as a function of Poisson’s ratio ν, shown in Figure 2.

Comparison of the obtained results with the data presented in [42] demonstrates their close agreement (Figure 2).

## 3. Results

**Figure 3:**
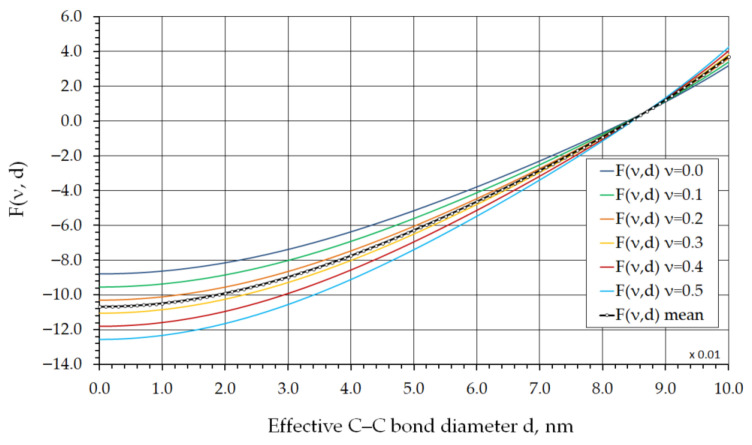
F(ν,d) plotted as a function of d for different values of Poisson’s ratio ν in the range from 0 to 0.5.

**Figure 4:**
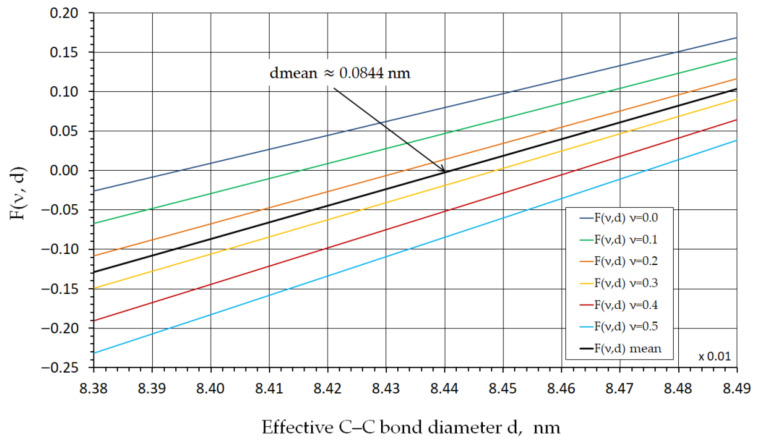
C–C bond diameter d as a function of Poisson’s ratio ν in the range from 0 to 0.5.

The results of the function $F\left(\nu ,d\right)\;$ in Equation (16), obtained by varying Poisson’s ratio ν in the range from 0 to 0.5, which corresponds to physically admissible values of this parameter for continuum materials, are shown in Figure 3 and Figure 4.

As can be seen from the graphs in Figure 3 and Figure 4, for Poisson’s ratio values within this range, the C–C bond diameter d varies from 0.08393 nm at ν=0 to 0.08474 nm at ν=0.5. The mean C–C bond diameter $d_{mean}\;$ is approximately 0.0844 nm at a Poisson’s ratio of ν=0.25.

The obtained numerical range of d for the C–C bond indicates that, when Poisson’s ratio is confined within physically admissible limits for continuum media, the effective diameter of the C–C bond remains nearly constant and exhibits only a weak dependence on ν. From the standpoint of structural mechanics, such behavior is expected because Poisson’s ratio primarily affects the redistribution of transverse shear stiffness, rather than longitudinal stiffness of the representative volume.

In this context, the obtained diameter can be interpreted as the lower-bound estimate of the effective transverse cross-section of the bond, which ensures equivalence between the molecular bending stiffness $k_{\theta }$ and the bending stiffness of a representative continuum element modeled within the Timoshenko framework. In contrast to the widely accepted nominal diameter of d = 0.147 nm, which is directly associated with the equilibrium spatial configuration of atomic orbitals, the present estimate represents a mechanically consistent characteristic derived strictly from energy-equivalence considerations. The presented correspondence between continuum and atomistic descriptions provides a fundamentally justified measure of the bond diameter, which prevents overestimation of flexibility under bending and avoids unphysical Poisson’s ratio values (e.g., ν > 0.5) that often arise when using simplified Euler–Bernoulli formulas.

Thus, for engineering calculations of nanostructures, such as carbon nanotubes, it is recommended to use the effective C–C bond diameter $d_{mean}=0.0844\;nm$, which, according to Equation (17), corresponds to a Poisson’s ratio of ν=0.25. This value of $d_{mean}$ may therefore serve as a reference geometric parameter in subsequent derivations of homogenized effective mechanical properties for periodic carbon-containing nanostructures (CNT arrays, graphene sheets, graphite laminates) under both static deformation and dynamic loading, including the propagation of waves at characteristic nanoscale lengths.

Let us consider the physical meaning of these results.

As demonstrated in [21], most chemical bonding concepts can be adequately described using the Electron Localization Function (ELF). The distribution of electron density is a fundamental characteristic of a molecule, determining its physical and chemical properties. The effective overlap region of the C–C σ-bond can be evaluated either by performing Density Functional Theory (DFT) calculations [20] or by analyzing molecular visualization moiré patterns [21].

**Figure 5:**
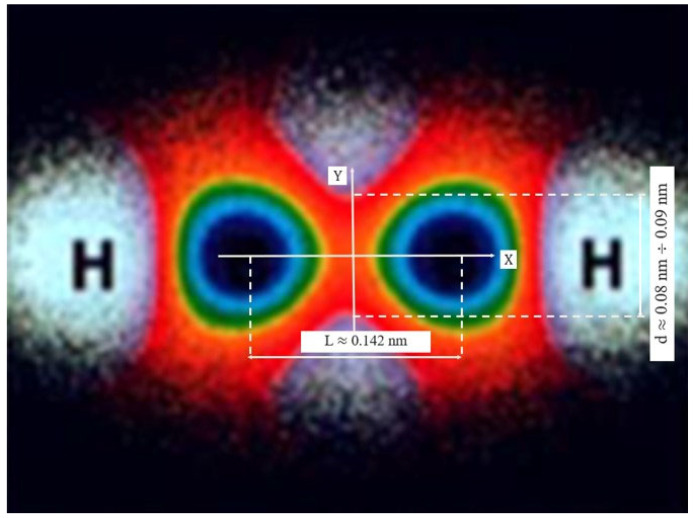
Two-dimensional moiré cross sections of the electron density distribution for the $C_{2}H_{2}$ molecule (extended Hückel method) [21].

The work [21] presents studies on the ELF distribution for various molecules and notes that the central positions of C atoms within molecular structures act as invariant attractors. This indicates that the topology of structurally invariant attractors (the centroids of structural elements) is always preserved regardless of the chemical correlation between neighboring groups (changes in bond angles or unit cell dimensions), as long as the number of primary neighbors around each invariant attractor remains unchanged [21]. Based on this, assuming that the distance between two carbon atoms is 0.142 nm, and by appropriately scaling the data shown in Figure 5, the effective diameter of the C–C bond’s electron density cross-section is found to vary in the range of approximately 0.08–0.09 nm. These estimates of the effective transverse width of the C–C σ-bond overlap are in good agreement with the results obtained from DFT calculations, which lie in the range of approximately 0.07–0.09 nm [20], as well as with the results presented above.

ELF calculations and experimental data indicate that the electron density in the C–C σ-bond region is highly concentrated around the bond axis, and the “characteristic bond width,” where the density remains high—e.g., at least 95% of the maximum—is approximately 0.1 nm in diameter. These findings are corroborated by numerous studies on ELF analysis for graphene and diamond [22].

To describe the transverse distribution of electron density, a smooth normal distribution is used, which adequately captures the symmetry and shape of the σ-bond electron cloud. The adopted model allows for the analytical calculation of electron density fractions as a function of the distance from the bond axis.

Assuming a Gaussian distribution for the function ρ(r) along the radius r perpendicular to the C–C bond axis, we can write:

(19) $$\rho (r)=\rho_{max}\;e^{\left(-\;\frac{r^{2}}{2\xi^{2}}\right)},$$

where r is the distance from the bond axis (transverse direction) in nm; $\rho_{max}$ describes the maximum density at the center, and ξ=0.015 nm is the effective width parameter of the distribution.

**Figure 6:**
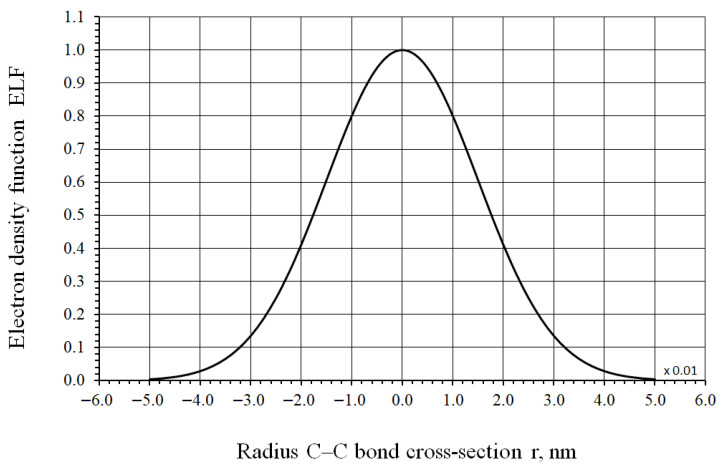
Normalized distribution of the electron density function (ELF) in the C–C bond cross-section.

Based on Equation (19), a normalized curve of the Gaussian electron density distribution (ELF), $F_{ELF}=\frac{\rho (r)}{\rho_{max}}=e^{\left(-\frac{r^{2}}{2\xi^{2}}\right)}$, along the radius (perpendicular to the bond axis) is obtained, as shown in Figure 6.

**Figure 7:**
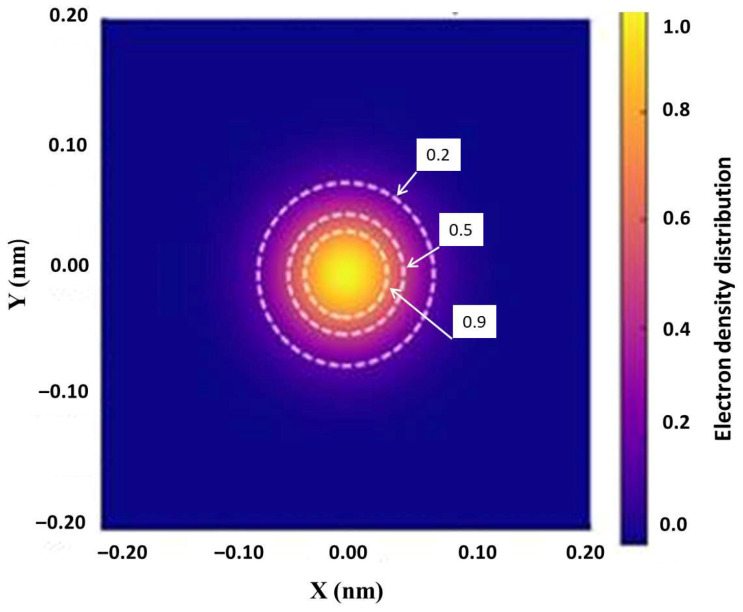
Moiré pattern of the electron density distribution in the transverse section of the C–C bond. White dashed lines indicate normalized density isolines at 0.9, 0.5, and 0.2 of the maximum value.

To estimate the radius of the region containing a specified percentage of the electron density, an integral calculation of the cumulative density is performed, assuming a cylindrical geometry for the bond. Based on the chosen profile, the radii corresponding to 20%, 50%, and 90% of the cumulative electron density are determined. These radii uniquely define the transverse size of the region of maximum electron localization. A 2D map of the electron density distribution in the transverse section of the C–C bond, corresponding to the Gaussian distribution given by Equation (19), is shown in Figure 7. Calculations show that the transverse radius of the region containing 90% to 99% of the electron density increases from 0.04 nm to 0.05 nm. The difference between these cumulative density levels reflects the exponential decay of electron density along the transverse radius of the bond.

Determining the transverse diameter of the bond’s electron cloud is crucial for refining the stress distribution in nanostructures, as the thickness of the electron density influences the calculation of bending and torsional stiffnesses when these values are incorporated into multiscale models for an accurate description of the mechanical behavior of carbon materials. Analysis of the electron density distribution curve indicates that the effective radius of the C–C bond lies within the range r≈0.04 nm÷0.045 nm, while the corresponding effective diameter falls within d≈0.08 nm÷0.09 nm.

The obtained value of the average C–C bond diameter, according to Equation (16), $d_{mean}=0.0844\;\mathrm{nm}$, is fully consistent with these data. The deviation from the average diameter ranges from –0.514% to 0.43%, which lies within the accuracy limits of the molecular mechanics force constants $k_{r},k_{\theta },k_{\tau }$, and the adopted shear coefficient $K=\frac{6(1+\nu )}{\left(7+6\nu \right)}$.

Given the known average value of the C–C bond diameter, the effective Young’s modulus of the C–C bond can be determined based on the generalized Morse potential $U\left(r\right)$, which represents a semi-empirical approximation of the true interatomic interaction potential and is expressed as [14]:

(20) $$U\left(r\right)=D_{e}\left[{\;(1-e^{-\alpha \left(r-r_{0}\right)})}^{2}-1\right]=D_{e}(e^{-2\alpha (r-r_{0})}-{2e}^{-\alpha (r-r_{0})}),$$

where $U\left(r\right)$ is the specific potential energy of the bond, $D_{e}$ = 0.6031 nN·nm is the bond dissociation energy between the atoms for hybridized bonds ${sp}^{2}$ [16], $\alpha =\sqrt{k_{r}/(2D_{e})}$ = 23.24 nm^−1^ is a parameter defining the curvature of the potential well, r is the interatomic distance (nm), and $r_{0}$ is the equilibrium bond length between the atoms.

In the case of bond deformation along the longitudinal axis, the strain component ε is given by

(21) $$\varepsilon =(r-r_{0})/r_{0},$$

and Equation (20) takes the form:

(22) $$U\left(\varepsilon \right)=D_{e}(e^{-2\alpha r_{0}\varepsilon }-{2e}^{-\alpha r_{0}\varepsilon }),$$

The physical, unnormalized components of the elastic strain tensor are determined according to the well-known relation [20] $C^{ijkl}$ = $\frac{\partial^{2}U\left(\varepsilon_{ij}\right)}{\partial \varepsilon_{ij}\partial \varepsilon_{kl}}{|}_{\varepsilon_{ij}=0,\varepsilon_{kl}=0}$, and the expression for calculating the elastic modulus is obtained after performing the normalization operation under the condition that $\varepsilon =0\;\mathrm{and}\;k_{r}={2D}_{e}{\left(\alpha \right)}^{2}\;$:

(23) $$E=\frac{1}{V}\frac{\partial^{2}U\left(\varepsilon \right)}{\partial \varepsilon \partial \varepsilon }{|}_{\varepsilon =0}=\frac{2D_{e}{\left(\alpha r_{0}\right)}^{2}}{V}=\frac{8D_{e}{\left(\alpha \right)}^{2}r_{0}}{\pi d^{2}}=\frac{4k_{r}r_{0}}{\pi d^{2}}\;$$

The shear modulus G of the representative volume of the C–C bond is determined according to the established relation:

(24) $$G=\frac{E}{2(1+\nu )}=\frac{4\;D_{e}{\left(\alpha r_{0}\right)}^{2}}{\pi d^{2}r_{0}(1+\nu )}\;=\;\frac{2\;{\;k}_{r}r_{0}}{\pi d^{2}(1+\nu )}\;,$$

**Figure 8:**
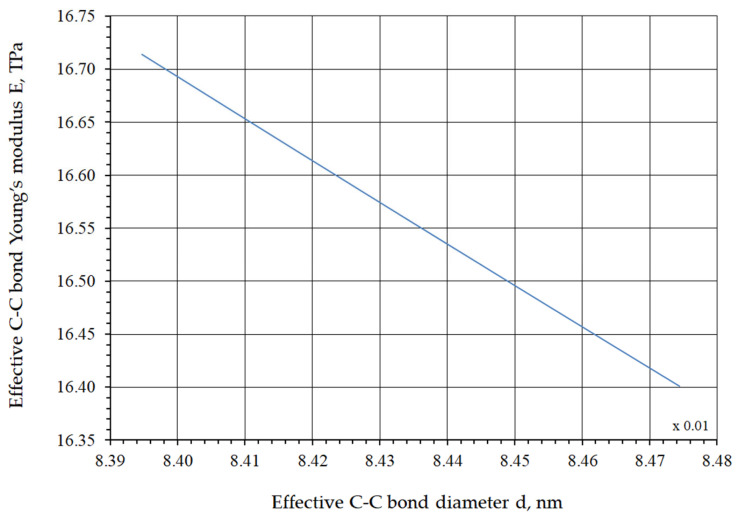
Dependence of the Young’s modulus on the C–C bond diameter.

**Figure 9:**
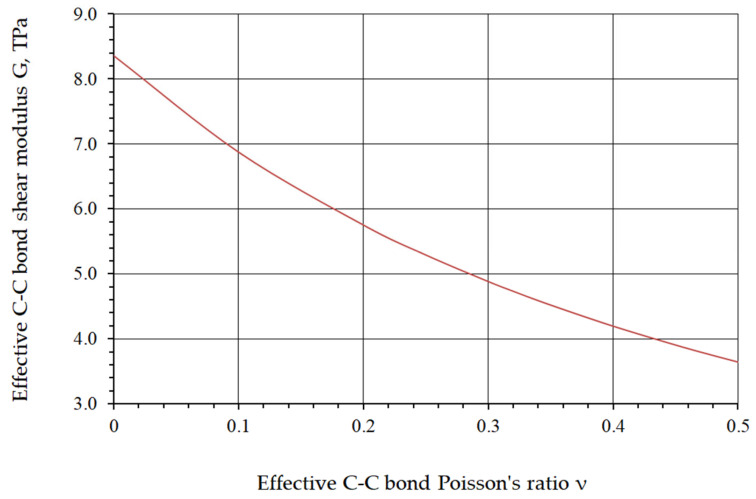
Dependence of the shear modulus G on the Poisson’s ratio ν of the C–C bond.

The functional dependencies of Young’s modulus on the C–C bond diameter and of the shear modulus on Poisson’s ratio are shown in Figure 8 and Figure 9, respectively.

## 4. Discussion

The obtained values of the transverse diameter of the C–C bond electron density localization region are essential for refining mechanical and multiscale models of carbon materials. In classical continuum models, which use the Timoshenko or Euler–Bernoulli beam approximations to describe the behavior of carbon nanostructures, the C–C bond is often treated as an element with equivalent mechanical properties, such as Young’s modulus, shear modulus, and geometric characteristics of its cross-section. However, this approach inherently assumes a simplified bond geometry and neglects the actual spatial distribution of electron density. Studies conducted within quantum-mechanical frameworks, in particular using Density Functional Theory (DFT), show that the electron density of the σ C–C bond exhibits a spatial distribution resembling a Gaussian profile elongated along the interatomic axis. The corresponding isolines exhibit a smooth exponential decay away from the bond center, as reported in [5]. Therefore, the effective cross-sectional area is governed not by atomic radii or tabulated parameters, but by the electron density distribution that determines bond strength and stiffness. Consequently, defining an effective cross-sectional diameter corresponding to a specified fraction of the electron density is essential for the accurate interpretation of the phenomenological elastic properties of a representative diatomic volume.

The value obtained in this study, $d_{mean}=0.0844nm$, corresponding to 90–99% of the bond electron density, ensures consistency between molecular-mechanical and continuum models. Accurate determination of the geometric parameters of the C–C bond is critical because bending stiffness depends on the moment of inertia (I∼d^4^), torsional stiffness depends on the polar moment (J∼d^4^), and both directly affect the coupling between bending and torsional modes.

Accounting for these factors is crucial when modeling the behavior of carbon nanotubes and graphene fragments. In particular, studies [38,46,47] have shown that errors in the chosen bond diameter of even 10–20% can lead to significant deviations in predicted stiffness values, especially for small-diameter nanotubes. Table 1 presents the values of the effective C–C bond thickness, Young’s modulus, and Poisson’s ratio used in the calculations of single-walled carbon nanotubes [2,41].

The elastic modulus $E_{c–c}$, listed in Table 1 corresponds to the effective elastic modulus of a continuum rod element at the C–C bond level in a carbon nanotube (CNT), determined according to Formula (23). The obtained value of $E_{c–c}$ characterizes the theoretical upper bound of stiffness for a covalent carbon beam.

To determine the effective elastic modulus of the carbon nanotube, E, this value must be normalized with respect to the effective CNT wall thickness, taken as the graphite interlayer spacing $t_{cnt}$ = 0.34 nm [1,2,48,51]: ${E=E}_{c–c}{\left(d_{c–c}/t_{cnt}\right)}^{2}=16.53{\left(0.0844/0.34\right)}^{2}=1.0186\;TPa$.

The obtained value of E_cnt is in good agreement with reported data for the effective elastic modulus of graphene and CNTs with different chiral indices (0.99–1.1 TPa) [1,2,16,48,51,52].

Thus, the difference between the “atomistic” (16.53 TPa) and “engineering” (0.99–1.1 TPa) elastic moduli arises from the scale at which the electron density of the C–C system is averaged.

The proposed approach provides a physically justified interpretation of the elasticity of carbon nanosystems as a manifestation of the energy density localized in the covalent bond core and distributed over the geometric volume of the structure.

In addition, accounting for the Timoshenko shear factor made it possible to establish a unique relationship between the elastic modulus, Poisson’s ratio, the diameter of the effective C–C bond rod element, and the molecular mechanics constants while satisfying the principle of thermodynamic stability.

Another advantage of this model is that, for $d_{c–c}$ = 0.0844 nm, solid finite elements (FE) may be used instead of beam elements, thereby expanding the capabilities of strength analysis for nanomodified materials within the finite element framework.

**Figure 10:**
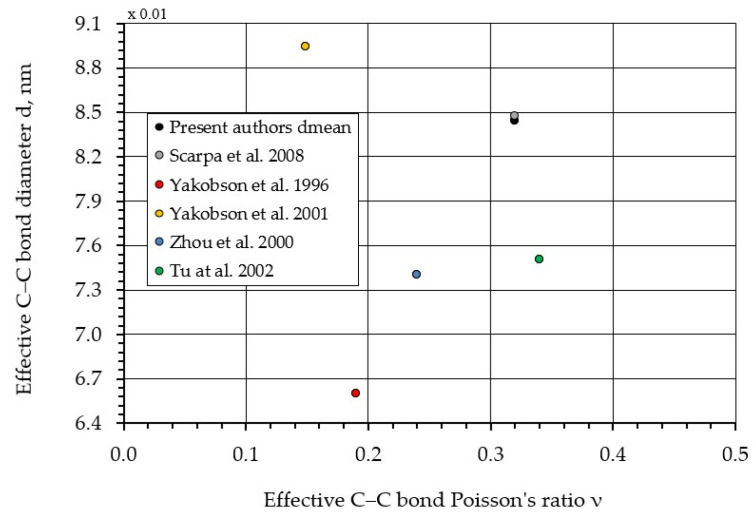
Comparison of the dependence of the C–C bond diameter $d_{mean}$ on Poisson’s ratio with results from other authors [38,42,48,49,50].

Figure 10 presents a comparison of the C–C bond diameter–Poisson’s ratio correspondence obtained in this work with results reported by other authors [38,42,48,50].

The reported Poisson’s ratio varies from 0.149 to 0.34 (Δ = 56%), whereas the C–C bond diameter ranges from 0.066 nm to 0.0896 nm (Δ = 26%). The relatively high discrepancies in these parameters lead to an even greater spread in the values obtained for the Young’s modulus (Δ = 61%) (Table 1).

This emphasizes the need for further refinement of models describing diatomic covalent interactions.

To verify the practical applicability of the proposed bond-level parameterization, the derived effective C–C bond characteristics were implemented within a finite element framework for the simulation of single-walled carbon nanotubes (SWCNTs). In contrast to purely analytical scaling estimates, the present results were obtained through direct numerical modeling of nanotube geometries with explicitly defined chiral indices.

Armchair (n, n) and Zigzag (n, 0) configurations were constructed according to their crystallographic definitions, with the atomic lattice mapped into an equivalent continuum representation using the previously determined effective bond diameter $d_{c–c}$ = 0.0844 nm and the corresponding elastic constants $E_{c–c}$, $G_{c–c}$, and Poisson’s ratio. Each C–C bond was modeled as a continuum rod element consistent with the Timoshenko beam formulation, ensuring compatibility between bending, axial, and shear deformations.

For each nanotube, the reduced Young’s modulus was obtained from finite element simulations under small-strain axial displacement loading conditions. The effective modulus E was evaluated from the linear stress–strain response by normalizing the total axial stiffness with respect to the effective wall thickness $t_{cnt}$ = 0.34 nm. This procedure ensured consistency with conventional definitions used in the literature and enabled direct comparison with previously published data.

Table 2 presents the reduced Young’s modulus values obtained for Armchair and Zigzag SWCNTs with various chiral indices and compares them with results reported by Tserpes et al. [2], Lu et al. [16], and Esbati et al. [52].

For Armchair nanotubes with indices ranging from (3, 3) to (20, 20), the predicted modulus remains nearly constant (~1.083–1.084 TPa), demonstrating weak diameter dependence and converging toward the graphene limit, in agreement with established theoretical expectations. For larger diameters (e.g., (25, 25)), a slight reduction is observed, reflecting geometric scaling effects inherent to the normalization procedure.

**Figure 11:**
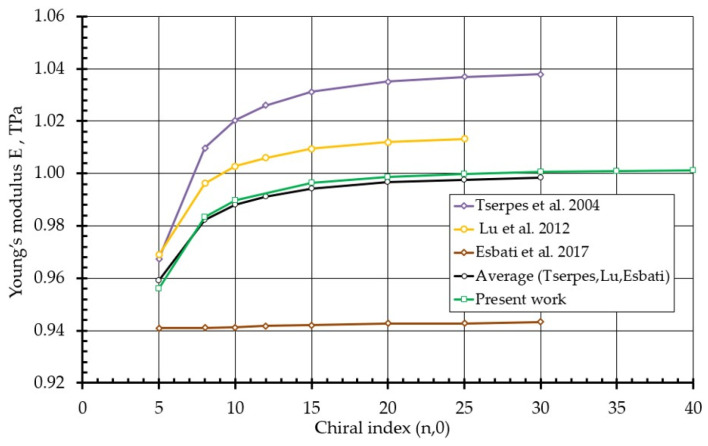
Variation of Young’s modulus on the chiral index for Zigzag-type single-walled carbon nanotubes [2,16,52].

For Zigzag nanotubes, the modulus exhibits moderate variation for small diameters ((5, 0)–(15, 0)), followed by asymptotic convergence toward ~1.0 TPa as the diameter increases (Figure 11). This behavior is consistent with curvature-induced stiffness reduction at small radii and gradual recovery toward the planar graphene limit.

The close agreement with previously reported values across a wide range of chiral indices confirms that the proposed electron-density-based bond diameter provides a mechanically consistent transfer of stiffness from the bond scale to the nanotube scale. Importantly, this agreement is achieved without empirical fitting at the nanotube level, thereby demonstrating the predictive capability of the developed multiscale framework.

## 5. Conclusions

In this study, the fundamental multiscale problem of correlating the atomistic stiffness characteristics of the C–C covalent bond with its mechanical representation at the continuum level has been addressed. It has been shown that the direct application of classical Euler–Bernoulli beam assumptions to molecular-mechanics-derived stiffness constants leads to internal inconsistencies. In particular, neglecting transverse shear deformation may result in nonphysical values of Poisson’s ratio (ν ≥ 0.5), violating the requirement of positive definiteness of the elastic strain energy.

To eliminate this inconsistency, a formulation based on Timoshenko beam theory has been adopted, providing an energetically consistent description of axial, bending, and torsional deformation modes of an individual C–C bond. By explicitly linking the molecular stiffness constants $k_{r}$, $k_{\theta }$, and $k_{\tau }$ to the structural parameters of an energetically equivalent representative-volume model, a sixth-order algebraic equation for the effective bond diameter $d_{mean}$, dependent on the Poisson’s ratio, has been derived.

The analysis of this equation demonstrates that the effective diameter exhibits only weak sensitivity to Poisson’s ratio within its physically admissible range 0 < ν < 0.5. Within this interval, full energetic compatibility between the atomistic description of the two-atom C–C system and its continuum analogue is preserved. Importantly, this same range corresponds to the condition of thermodynamic stability of the equivalent continuum, ensuring the positive definiteness of the elastic energy and excluding mechanically inadmissible states.

It is emphasized that the ambiguity addressed in this work does not originate from a particular choice of interatomic potential. Regardless of whether atomistic stiffness parameters are obtained from classical force fields, reactive bond-order potentials, quantum-chemical calculations, or analytically tractable anharmonic models such as the Morse potential adopted here, the transition to a structural-mechanical representation inevitably introduces an additional geometric parameter—the effective bond diameter. In the present study, the Morse functional serves as a physically interpretable and analytically transparent reference model, allowing a controlled derivation of bond stiffness and an explicit analysis of the multiscale mapping procedure itself.

The proposed method may be directly employed in multiscale mechanical modeling of carbon-based nanostructures, including graphene sheets, carbon nanotubes, and nanomodified composites reinforced with graphene or CNT inclusions. It is particularly relevant in situations where direct molecular dynamics simulations are computationally impractical, for example when large representative volume elements are required or when structural components extend to the micro- or macroscale.

The approach is especially applicable when a continuum finite element (FE) model must be constructed while maintaining strict consistency between its constitutive parameters and the underlying atomistic physics. This is crucial in modeling frameworks that employ three-dimensional structural analogues of atomic lattices, where effective bond-level parameters directly govern the macroscopic elastic response of the material.

The methodology is intended for researchers engaged in the development of graphene- or carbon nanotube-based nanomodified materials; specialists designing systematic transition schemes from atomistic to continuum modeling; experts in multiscale mechanics of nanomaterials; and engineers constructing three-dimensional finite element models of graphene- and CNT-reinforced materials that require energetically consistent and physically grounded bond-level parameters.

Overall, the proposed formulation provides a rigorous and transferable foundation for multiscale mechanical modeling of carbon-based materials, ensuring that continuum-level simulations remain firmly anchored to atomistic energetics and to the fundamental requirements of mechanical and thermodynamic stability.

## Figures and Tables

**Table 1:** Typical values of Young’s modulus, Poisson’s ratio ν, and effective wall thickness d of the effective C–C bond for the single-walled carbon nanotubes reported by different authors.

Authors	Method	Young’s Modulus, TPa	Poisson’s Ratio ν	d, nm
Yakobson et al. [38]	Molecular dynamics Tersoff-Brenner potential	5.5	0.19	0.066
Yakobson et al. [48]	Molecular dynamics	3.859	0.149	0.0894
Lu [47]	Molecular dynamics The force-constant model	0.97	0.28	0.34
Zhou et al. [49]	Tight-binding model	5.1	0.24	0.074
Kudin et al. [39]	Ab inito computations	3.859		0.0894
Tu et al. [50]	Local density approximation model	4.7	0.34	0.075
Pantano et al. [51]	Continuum shell Modeling	4.84		0.075
Tserpes et al. [2]	Structural mechanics: FE method	5.49		0.147
Present authors $E_{c–c}$	Structural mechanics: Morse potential	16.53	0.25	0.0844

**Table 2:** Reduced Young’s modulus of SWCNT.

SWCNT Type	Chiral Index (*n*, *m*)	Reduced Young’s Modulus E, TPa			
		Tserpes et al. [2]	Lu et al. [16]	Esbati et al. [52]	Present Work
Armchair	(3, 3)	1.0381	1.0181	–	1.082
	(5, 5)	1.0377	1.0167	–	1.083
	(10, 10)	1.0379	1.0165	–	1.083
	(12, 12)	1.0379	1.0166	1.019	1.083
	(15, 15)	1.0381	1.0167	1.013	1.084
	(20, 20)	1.0382	1.014	–	1.084
	(25, 25)	1.0369	1.0133	0.9428	0.9976
Zigzag	(5, 0)	0.9674	0.9689	–	0.9559
	(8, 0)	1.0098	0.9962	–	
	(10, 0)	1.0204	1.0028	0.9412	0.9899
	(15, 0)	1.0312	1.0095	0.942	0.9964
	(20, 0)	1.0351	1.0120	0.9427	0.99875
	(25, 0)	1.0369	1.0133	0.9428	0.9976
	(30, 0)	1.0379	–	0.9433	1.0005
	(35, 0)	–	–	–	1.0008
	(40, 0)	–	–	–	1.0012

## Data Availability

No new data were created or analyzed in this study. Data sharing is not applicable to this article.

## Abbreviations

The following abbreviations are used in this manuscript:

C–C	Covalent bond between two carbon atoms
$F_{ELF}$	Gaussian distribution of the electronic-density function in the transverse cross-section of the C–C bond
R	Generalized coordinate in the radial direction of the C–C bond’s transverse cross-section, nm
d	Effective bond diameter, nm
$d_{mean}$	Average effective bond diameter, nm
Ν	Effective Poisson’s ratio of the bond
$k_{r}$	Force constant of molecular mechanics (tension), $\frac{nN}{nm}$
$k_{\theta }$	Force constant of molecular mechanics (bending), $\frac{nN\;nm}{{rad}^{2}}$
$k_{\tau }$	Force constant of molecular mechanics (torsion), $\frac{nN\;nm}{{rad}^{2}}$
$D_{e}$	Bond dissociation energy between the atoms, J
α	Parameter defining the curvature of the potential well, nm^−1^
I	Moment of inertia, nm^4^
J	Polar moment of inertia, nm^4^
ELF	Electron Localization Function
F(ν,d)	Residual function
K	Shear coefficient
$U_{A},U_{M},U_{\tau }$	Potential energy of the beam under tension, bending, and torsion, J
Φ	Sheardeformationconstant
E	Young’s modulus, TPa
G	Shear modulus, TPa
A	Cross-sectional area, nm^2^
L	Bond length, nm
∆L	Bond elongation, nm
α	Bond bending angle increment, rad
∆β	Bond torsion angle increment, rad
